# Galectin-1 Overexpression in Endometriosis and Its Regulation by Neuropeptides (CRH, UCN) Indicating Its Important Role in Reproduction and Inflammation

**DOI:** 10.1371/journal.pone.0114229

**Published:** 2014-12-04

**Authors:** Aikaterini Vergetaki, Udo Jeschke, Thomas Vrekoussis, Eirini Taliouri, Luca Sabatini, Evangelia A. Papakonstanti, Antonis Makrigiannakis

**Affiliations:** 1 Department of Obstetrics and Gynecology, Medical School, University of Crete, Heraklion, Greece; 2 Department of Obstetrics and Gynecology, Innenstadt campus, Ludwig Maximilians University of Munich, Munich, Germany; 3 Department of Obstetrics and Gynecology, Medical School, University of Ioannina, Ioannina, Greece; 4 Centre for Reproductive Medicine, St Bartholomew’s Hospital, London, United Kingdom; 5 Department of Biochemistry, Medical School, University of Crete, Heraklion, Greece; Rutgers University, United States of America

## Abstract

Endometriosis is an inflammatory disease of women of reproductive age featured by the presence of ectopic endometrium and is strongly related to infertility. Galectins, carbonhydrate-binding proteins, have been found to have pro- or anti-inflammatory roles in the reproductive tract and in pathological conditions concerning infertility. Galectin-1, which is expressed at endometrium and decidua, plays a major role in implantation and trophoblast invasion. Also, the neuropeptides, corticotropin releasing hormone (CRH) and urocortin (UCN) and their receptors are expressed in eutopic and ectopic endometrium showing a differential expression pattern in endometriotic women compared to healthy ones. The aim of this study was to examine the galectin-1 expression in endometriotic lesions and compare its expression in eutopic endometrium of endometriotic and healthy women. Furthermore, we examined the effect of CRH and UCN in galectin-1 expression in Ishikawa cell line and macrophages and investigated the implication of CRHR1 in these responses. Eutopic and ectopic endometrium specimens, Ishikawa cell line and mice macrophages were used. Immunohistochemistry and western blot analysis were performed in order to identify galectin-1 expression in ectopic and eutopic endometrium of women with and without endometriosis and the regulatory effect of CRH and UCN on galectin-1 expression. This study presents for the first time that galectin-1 is overexpressed in endometriotic lesions compared to eutopic endometrium of endometriotic women and is more abundantly expressed in eutopic endometrium of disease women compared to healthy ones. Furthermore, it is shown that CRH and UCN upregulate galectin-1 expression in Ishikawa cell line and macrophages and this effect is mediated through CRHR1. These results suggest that galectin-1 might play an important role in endometriosis pathology and infertility profile of women suffering from endometriosis by being at the same time regulated by CRH and UCN interfering in the immune disequilibrium which characterizes this pathological condition.

## Introduction

Endometriosis is a benign chronic disease affecting 10% of women of reproductive age. Endometriosis, featured by stromal and epithelial ectopic endometrial tissue [1]–[5] is accompanied with adhesion formation, pelvic pain and infertility. Although its pathogenesis has not been completely identified, endometriosis has been characterised as an estrogen-dependent chronic inflammatory disease [6]–[8]. Endocrine/paracrine influences and immunological aspects have also been linked to this disease. In addition, several growth factors, cytokines, immune cells and hormones have been proposed as involved in the pathophysiology of endometriosis-related infertility, via altering both the eutopic and the ectopic endometrium [3], [9].

Galectins are carbonhydrate-binding proteins which bind b-galactoside [10]. Galectins have strongly been implicated in inflammation, autoimmunity and cancer and have been thought to be useful anti-inflammatory targets. As far as the immune system is concerned, galectins are important regulators of activated macrophages and activated B cells. They also play a vital role in T cell homeostasis and survival. Galectins are widely expressed by reproductive tissues. Galectin-1, -3, -9, -15 have been found to be expressed in human endometrium and decidua, while galectin-1(gal-1) has been also found to be expressed by endometrial stromal cell in humans [11]–[13] . Galectins have been reported as potential contributors to endometrial immune system regulation; galectins seem to play a vital role in leukocyte regulation by interfering into cell adhesion, migration and chemotaxis [14]. Interestingly, decidual expression of gal-1 is regulated by progesterone, while galectin-1 increases progesterone concentrations. This positive feedback between galectin-1 and progesterone is considered of great importance in pregnancy maintenance [15]. Gal-1 expression levels in endometrium vary during the menstrual cycle, being increased significantly at late secretory phase of endometrium and in the decidua. At the same time a very interesting gal-1 expression pattern in trophoblastic tissue has been shown [13]. Gal-1 is also synthesized, prior to implantation, in the trophoectoderm of blastocysts and has also been recently found to participate in human trophoblast cell invasion machinery [16]. It is thus clear, that gal-1 is an important contributor to a successful pregnancy.

As endometriosis is characterized by the expression of several proteins including growth factors, integrins, cadherins and lectins that regulate cell migration, invasion, angiogenesis, immune functions and apoptosis, galectins are expected to play an important role in this inflammatory disease. So far, only galectin-3 has been found to be overexpressed in various forms of endometriosis compared to eutopic endometrium of women with endometriosis and to be more highly expressed in the eutopic endometrium of women with endometriosis compared to the eutopic endometrium of women without endometriosis [17]. Taking into consideration that gal-1 has a vital role at inflammatory sites, in implantation and decidualization as well, combined with the fact that endometriosis is accompanied with inflammation and infertility, we thought that it would be interesting to investigate and compare gal-1 expression in eutopic endometrium of both healthy and disease women and to compare gal-1 expression levels in eutopic and ectopic endometrium of disease women trying to further clarify gal-1 role in endometriosis.

Corticotropin Releasing Hormone (CRH) is a 41-amino acid neuropeptide, synthesised in the hypothalamus, regulating the hypothalamus-pituitary-adrenal axis [18], [19]. CRH expression and biological functions are mediated by its membrane receptors, CRH-R1 (α, β, γ, c-h) and CRH-R2 (α, β, γ) [20]–[22]. CRH receptors are also activated by other endogenous agonists, such as urocortin (UCN), which is a 40-amino acid peptide belonging to the corticotropin-releasing hormone family and is structurally related to CRH [23]–[25]. Apart from the central nervous system, CRH and its receptors are expressed in several sites of the female reproductive system, including the endometrial glands, decidualized stroma, trophoblast, syncytiotrophoblast and placental decidua [26]–[28]. Moreover, CRH and UCN are secreted at inflammatory sites, acting as proinflammatory factors [18]–[21], [23], [24], [29], [30]. Reproductive CRH has been shown to serve as an autocrine and paracrine modulator and to participate in decidualization, embryo implantation and maintenance of human pregnancy [27], [31]–[39]. In addition, CRH and UCN mRNA have been found to be expressed by endometriotic cells, while endometriotic lesions show a strongly positive staining reaction for CRH and UCN [40]. The expression and function of CRH and UCN have also been found to be impaired in eutopic endometrium of women with endometriosis [41]. Moreover, we have recently shown that CRH and UCN receptor subtypes CRHR1b and CRHR2a are expressed in endometriotic sites and that they are more strongly expressed in eutopic endometrium of women with endometriosis compared to healthy women endometrium at mRNA and protein level. CRH, UCN, CRHR1 and CRHR2 mRNA were also more highly expressed in ectopic rather than eutopic endometrium in women with endometriosis. These data indicate that CRH and UCN might play an immunoregulatory role in endometriotic sites [42]. So, it would be of quite interest to investigate if and how these neuropeptides regulate proteins (such as galectins) participating in fetal-maternal crosstalk and inflammatory pathological situations such as endometriosis.

Considering all the above, the aim of this study was i) to identify the gal-1 expression in the eutopic and the ectopic endometrium of women with endometriosis and compare its expression with the eutopic endometrium of healthy women and ii) to examine whether and how CRH and UCN regulate gal-1 expression in eutopic endometrium and macrophages.

## Materials and Methods

### Tissue sample collection

Biopsy specimens of endometrium (at proliferative, early secretory and secretory phase, confirmed by the progesterone levels of the women) were taken from healthy women (10 patients) undergoing hysteroscopy for diagnostic reasons due to spontaneous spotting haemorrhage. When comparing protein expression levels in eutopic endometrium of healthy and disease women, only eutopic endometrium at the secretory phase was used. They were all healthy apart from 3/10 having small polyps, as their hysteroscopy results showed. Eutopic and ectopic endometrial tissue biopsies (stage III and IV) at secretory phase, as it was confirmed by the progesterone levels of the patients, were obtained from 16 patients diagnosed with endometriosis on different sites (peritoneal nodule, rectovaginal nodule, rectouterine nodule, right and left ovarian cyst endometriosis, left and right uterosacral legiment nodule), sharing all the same pathology, in the Department of Obstetrics and Gynaecology, St Bartholomew’s Hospital of Queen Mary University, London, UK (Research Ethics Committee Reference Number: 05/Q0604/44). This research protocol was approved by the Ethics Committee of Queen Mary University, London, UK. All participants provided their written informed consent to participate. It is important to notice that the most critical reproductive hormone levels of both healthy and endometriotic patients did not affect the outcome of our research protocols as they ranged among: FSH levels (day 3 of the menstrual cycle): healthy patients 6–8 mlU/ml, endometriotic patients 7–9 mlU/ml, E_2_ levels (day 3 of the menstrual cycle): healthy patients 45±7 pg/ml, endometriotic patients 50±11 pg/ml and Progesterone levels (day 21 of the menstrual cycle): healthy patients 17±2 ng/ml, endometriotic patients 15±3 ng/ml. In addition, all the detailed inclusion and exclusion criteria taken into consideration for tissue sample collection are the following. Inclusion criteria for tissue sample collection: i) Reproductive aged women, ii) normal steroid hormone profile, iii) women undergoing hysteroscopy for diagnostic reasons due to spontaneous spotting haemorrhage, iv) stage III and IV endometriosis diagnosed during surgery. Exclusion criteria for tissue sample collection; i) women that have received hormonal treatment at least three months prior to surgery, ii) women under treatment for other pathologies, iii) women with possible endometrial hyperplasia, iv) women suffering from other endometrial pathologies diagnosed during hysteroscopy.

### Cell culture

#### Ishikawa cell line culture

Ishikawa cell line: Human endometrial adenocarcinoma cell line. Ishikawa cells show characteristics of glandular and luminal epithelium and express many of the enzymes, proteins and steroid receptors present in normal endometrium, which makes them suitable for the study of endocrine signaling in endometrium. Ishikawa cells are widely considered a good model for studying endometrium function. Ishikawa cell line was a generous gift from Prof Udo Jeschke, bought from the European Collection of Cell Cultures (ECACC, Salisbury, UK). Ishikawa cell line was cultured in DMEM Glutamax supplemented with 10% FBS (inactivated at 56°C for 1 hr), 1% Penicilline/Streptomysin, 1% Sodium Pyruvate and 1% Anti – Mycotic at a humidified incubator in a 5% CO_2_ atmosphere at 37°C. The medium used to be changed every 48 hrs.

#### Isolation and culture of murine bonne marrow macrophages (BMMs)

BMMs were derived from at least three 6 to 8 week old mice per experiment. Cells were cultured on bacteriological plastic plates at 10^6^ cells/ml in macrophage growth medium consisting of RPMI 1640 (GIBCO-Invitrogen Ltd., Paisley, UK), 1 mM sodium pyruvate (GIBCO), 1x non-essential amino acids (GIBCO), 0.029 mM 2-mercaptoethanol (Sigma), 10% heat-inactivated FCS (GIBCO) supplemented with 10% L cell-conditioned medium as a source of CSF-1. After 3 days, non-adherent cells were collected and either cryogenically stored in FBS containing 10% DMSO or cultured at 6–8×10^5^ cells/ml on petril dishes and cultured for 4 days before use. Cells were detached using EDTA, centrifuged at 1000×g, resuspended in macrophage growth medium and cultured for the experiments. All results were obtained from cells that had been cultured for no longer than 10 days after dissection. In all experiments, the medium was changed to macrophage starvation medium (macrophage growth medium without L-cell-conditioned medium) 16–20 h prior to experiments.

#### Cells incubated with peptides

Cells were incubated with the appropriate peptide according to each experimental protocol, before the protein lysate extraction protocol. CRH peptide (Tocris R&D Systems), stock of 10^−4^ M: 10^−7^ M final concentration in cells - medium. UCN peptide (Sigma-Aldrich), stock of 10^−4^ M: 10^−7^ M concentration in cells – medium. The incubation of cells with CRH and UCN peptide was for 0, 2, 8, 24 hrs. Antalarmin, stock of 10^−3^ M: 10^−6^ M final concentration in cells – medium together with CRH peptide and incubation of cells for 8, 24 hrs.

### Cell protein lysates extraction

Protein lysate has been extracted from Ishikawa cell line and mice macrophages according to the following protocol: After cells had been treated with the appropriate peptides added with 1x PBS. General Lysis Buffer (50 mM Tris, pH 7.5, 1 mM EDTA, 150 mM M NaCl, 1% Igepal, 50 mM NaF and οn day of cell lysis the following inhibitors: 1∶1000 of stock leupeptin 10 Mm, 1∶100 of stock 100 mM PMSF (in isopropanol), 10 µg/ml aprotinin, 1∶100 of stock 100 mM Na4V03 were added) was added cells were scabbed and placed in a tube on ice for 20 min by continuous vortexing. Cells were then centrifuged at 13000 rpm for 5 min. The supernatant at this step is the protein lysate. The whole quantity was removed in a new tube and placed on ice till used. The protein concentration was determined using Bradford protein assay method and a spectrophotometer, measuring proteins at 495 nm. Equal quantities of proteins had been used for western blotting analysis. 100 µg of protein is mixed with 4x sample buffer (Tris, b-mercaptoethanol, SDS, glycerol, 0.008 gr BPB) and boiled at a heat blocker at 95°C for 5 min. Samples were stored at −20°C, till to be used for western blotting analysis.

### Tissue protein extraction

Firstly, the T-PER (Thermoscientific) mix by adding inhibitors (as described above) was prepared and 1, 5 ml of T-PER mix was added in 0.100 gr of tissue for homogenization. After that, centrifugation at 10.000 g for 10 min followed. The supernatant at this step is the protein lysate. The supernatant was removed very carefully in new tubes and placed on ice till used. The protein concentration was determined using Bradford protein assay method and a spectrophotometer, measuring proteins at 495 nm. Proteins are mixed with Biorad (1 ml Biorad−1 Biorad: 5 water for injection H_2_O−+5 µl of protein extract) are measured at 495 nm. Equal quantities of proteins had been used for western blotting analysis. 100 µg of protein is mixed with 4x sample buffer (Tris, b-mercaptoethanol, SDS, glycerol, 0.008 gr BPB) and boiled at a heat blocker at 95°C for 5 min. Samples were stored at −20°C, till to be used for western blotting analysis.

### Western blotting analysis

100 µg of proteins were extracted from Ishikawa cell line, macrophages, healthy women’s eutopic endometrium (10 patients) and eutopic endometrium and endometriotic tissue from endometriotic patients (16 patients), followed by SDS-PAGE analysis in 10% acrylamide gel, and electrotransfer onto a nitrocellulose membrane. The membrane was blocked in 5% skim milk powder in 0.1% Tris-buffered saline/Tween for 20 min. The membrane was then incubated with galectin-1 (rabbit polyclonal anti-galectin-1 antibody, Abcam) at a dilution of 1∶1000, followed by incubation goat anti-rabbit IgG AP132P (Chemicon International, multipore). GAPDH (rabbit GAPDH antibody 14C10, Cell signalling) was used as a house keeping gene. Band intensities of protein of interest were normalized with band intensities of GAPDH and expressed as arbitrary units (a.u.).

### Immunohistochemical analysis

Formalin-fixed, paraffin-embedded tissue sections (4 µm thick) of eutopic endometrium from 16 patients were deparaffinized in xylene and rehydrated through graded concentrations of ethanol. Antigen retrieval (350W, 3 cycles, 5 min each in citrate buffer: 10% citric acid mix−9 ml citric acid and 41 ml sodium citrate in 450 ml ddH_2_0) was followed. After inhibition of endogenous peroxidases with 3% H_2_O_2_ (5 min), unspecific antibody binding was blocked with 10% power block (BioGenex Lig DAB substrate Pack, BioGenex Laboratories Inc, Fremont, CA, USA) for 10 min. Serial sections were then incubated with primary antibody against human galectin-1 (rabbit polyclonal anti-galectin-1 antibody, Abcam). Both blocking as well as detection and visualization of staining were performed by using the BioGenex Supersensitive link-label Detection System (BioGenex Laboratories Inc, Fremont, CA, USA) followed by the BioGenex Lig DAB substrate Pack (BioGenex Laboratories Inc, Fremont, CA, USA), according to the manufacturer’s protocols. Finally the slides were counterstained with Mayer’s heamatoxylin (Dako, Carpinteria, CA, USA) for 3 min, washed in tap water and covered using Glycergel (Dako, Carpinteria, CA, USA). Negative controls were performed by replacing the primary antibody with normal rabbit IgG as isotype control. The sections were examined by light microscopy. The intensity and distribution of the staining reaction were evaluated by two blinded, independent observers, including a gynecological pathologist, using the semiquantitative immunoreactive score (IRS). The IRS was calculated by multiplication of optical staining intensity including glandular and stromal staining (graded as 0 = no reaction, 1 = weak, 2 = moderate and 3 = strong staining) and the percentage of positive-stained cells (0 = no positive, 1 = <25% of the cells, 2 = 25–50% of the cells, 3 = 51–75% of the cells and 4 = >75% of the cells). The IRS score derived from both the glandular and the stromal staining of the tissues.

### Evaluation and statistical analysis

Western blot bands were analysed via image analysis software (Scion Corporation, Release Beta 4.0.2, Frederick, MD, USA). Statistical analysis was performed using the unpaired two-tailed Student’s *t*-test. Any statistical difference at p<0.05 was considered significant.

## Results

### 1. Galectin-1 is most highly expressed at the late secretory phase of eutopic endometrium of healthy women

**Figure 1:**
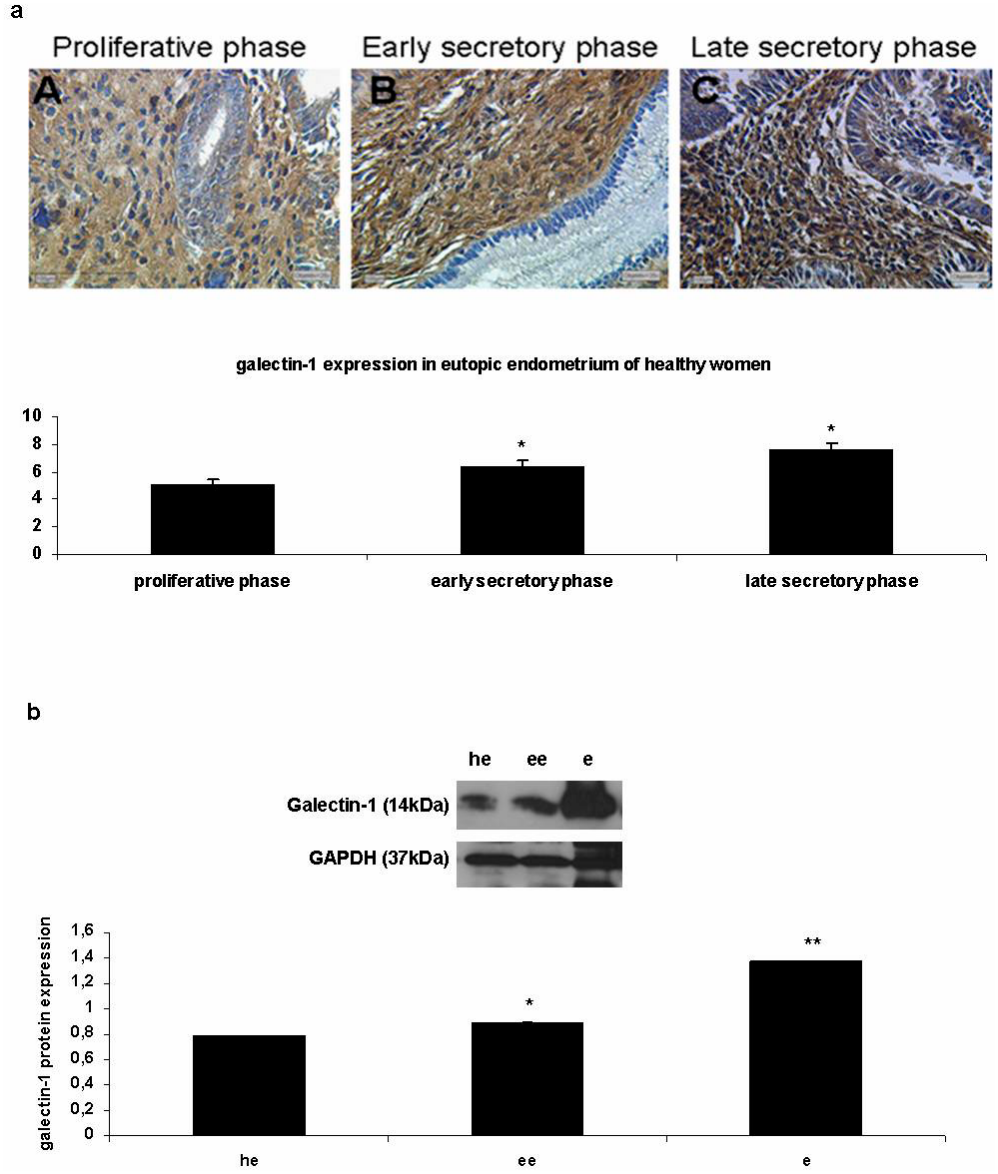
Galectin-1 overexpression in late secretory phase of eutopic endometrium and in endometriotic sites. **1a:** Immunohistochemical expression of galectin-1 expression through the different phases of the menstrual cycle of the endometrium (A: proliferative, B: early secretory phase, C: late secretory phase) showing a higher expression pattern at the late secretory phase of the endometrium(C), *p<0.05. **1b:** Western blot immunodetection of galectin-1 (14 kDa) protein expression in eutopic and ectopic endometrium. Galectin-1 is overexpressed in eutopic endometrium of endometriotic women (ee) when compared to eutopic endometrium of healthy women (he), *p<0.05 and galectin-1 shows an abundant expression in ectopic endometrium (e) when compared to eutopic endometrium of the same endometriotic women (ee), **p<0.01. GAPDH (37 KDa) was used as a house keeping gene.

In order to investigate the galectin-1 expression in eutopic endometrium of healthy women immunohistochemical analysis in eutopic endometrial tissue sections of healthy women (10 patients) was performed in three different phases of the menstrual cycle (proliferative, early secretory and late secretory phase). As shown in figure 1, galectin-1 expression is higher at late secretory phase (Figure 1a). According to IRS Score calculations galectin-1 expression is higher at the late secretory phase (proliferative phase: 5.125±0.25 a.u, early secretory phase 6.375±0.43 a.u, late secretory phase: 7.6875±0.38 a.u., p<0.05) and it is shown a 1.24 fold of gal-1 expression at early secretory phase endometrium when setting the proliferative phase endometrium as control and 1.20 fold of gal-1 expression at the late secretory phase when setting the early secretory phase endometrium as control.

### 2. Galectin-1 overexpression in endometriosis (ectopic endometrium)

In order to investigate the galectin-1 expression levels in eutopic endometrium of healthy women (n = 3), in eutopic and ectopic endometrium of endometriotic women (n = 3), western blotting (Figure 1b) was performed. When performing western blotting, it was shown that galectin-1 is overexpressed in ectopic endometrium (e) compared to eutopic endometrium of endometriotic women (ee) and eutopic endometrium of healthy women (he) (Figure 1b). Concerning Western blotting analysis: Galectin-1: 0.887±0.007 a.u in eutopic endometrium of endometriotic women vs 0.786±0.005 a.u in eutopic endometrium of healthy women, 1.12 fold increase, p<0.05, when setting eutopic endometrium of healthy women as control. Galectin-1: 1.378±0.003 a.u in ectopic endometrium vs 0.887±0.007 a.u in eutopic endometrium of endometriotic women, 1.55 fold increase, p<0.01, when setting eutopic endometrium of endometriotic women as control.

### 3. CRH and UCN upregulate galectin-1 expression

#### 3i. Upregulation of galectin-1 expression in Ishikawa cell line

**Figure 2:**
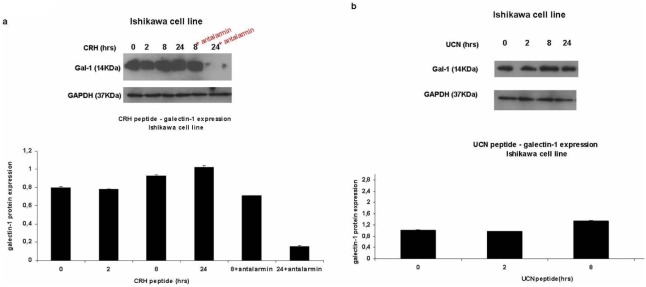
Galectin-1 is upregulated in Ishikawa cell line upon CRH and UCN stimulation and this is mediated through CRHR1. Western blot immunodetection of galectin-1 (14 kDa) protein expression in Ishikawa cell line, regulated by CRH and UCN neuropeptides. GAPDH (37 KDa) was used as a house keeping gene. **2a:** In Ishikawa cell line, galectin-1 expression is upregulated by CRH, showing a higher expression at 8 hrs and 24 hrs of stimulation. When antalarmin is added, galectin-1 shows an impaired expression mostly at 24 hrs of stimulation. **2b:** In Ishikawa cell line, galectin-1 is upregulated by UCN, showing a higher expression at 8 hrs of stimulation.

In order to investigate how galectin-1 expression is regulated in Ishikawa cell line upon stimulation with CRH peptide, western blotting was performed. Cells where treated with CRH peptide for 0, 2, 8, 24 hrs. Antalarmin, which is a non-peptide, synthetic, exogenous antagonist of CRHR1 [43], had been used at the time points where higher expression of galectin-1 was shown (8 hrs and 24 hrs of CRH peptide+Antalarmin). As a result, it was shown that CRH upregulated galectin-1 expression in Ishikawa cell line (Figure 2a) mostly at 8 hrs and 24 hrs of stimulation, time dependently and this was mediated by CRHR1. Antalarmin blocked the upregulative effect of CRH on galectin-1 expression mostly at 24 hrs of stimulation in Ishikawa cell line. (Galectin-1 expression in Ishikawa cell line – CRH peptide stimulation for 0 hrs: 0.8±0.007, 2 hrs: 0.78±0.004 a.u, 8 hrs: 0.9±0.006 a.u, 24 hrs: 1.02±0.018 a.u, 8 hrs+antalarmin: 0.7±0.004, 24 hrs+antalarmin: 0.15±0.009). After showing that CRH had an upregulative effect on galectin-1 expression in Ishikawa cell line, UCN peptide effect on galectin-1 expression had been tested at the same time points of stimulation (0, 2, 8, 24 hrs) in the same cell type. UCN peptide caused an increase in galectin-1 expression mostly at 8 hrs of stimulation in Ishikawa cell line (Figure 2b). (Galectin-1 expression in Ishikawa cell line – UCN peptide stimulation for 0 hrs: 1.01±0.006, 2 hrs: 0.97±0.003 a.u, 8 hrs: 1.33±0.02 a.u, 24 hrs: 0.88±0.03 a.u).

#### 3ii. Upregulation of galectin-1 expression in macrophages

**Figure 3:**
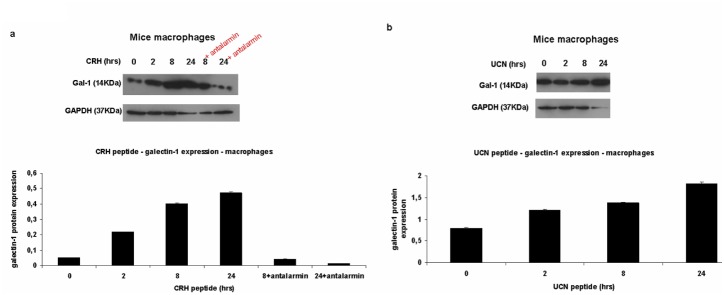
Galectin-1 is upregulated in macrophages upon CRH and UCN stimulation and this is mediated through CRHR1. Western blot immunodetection of galectin-1 (14 kDa) protein expression in macrophages, regulated by CRH and UCN neuropeptides. GAPDH (37 KDa) was used as a house keeping gene. **3a:** In mice macrophages, galectin-1 expression is upregulated by CRH mostly at 24 hrs and Antalarmin effect is stronger at 24 hrs. **3b:** In mice macrophages, galectin-1 expression is mostly upregulated by UCN at 24 hrs.

In order to investigate how galectin-1 expression is regulated in macrophages upon stimulation of these cells with CRH peptide, western blotting was performed. Cells were treated with CRH peptide for 0, 2, 8, 24 hrs. Antalarmin, which is a non-peptide, synthetic, exogenous antagonist of CRHR1, had been used at the time points where higher expression of galectin-1 was shown (8 hrs and 24 hrs of CRH peptide+Antalarmin). As a result, it was shown that CRH upregulated galectin-1 expression in macrophages mostly at 24 hrs of stimulation (Figure 3a) time dependently and this was mediated by CRHR1. Antalarmin blocked the upregulative effect of CRH on galectin-1 expression in a higher way at 24 hrs of stimulation in macrophages. (Galectin-1 expression in macrophages - CRH peptide stimulation for 0 hrs: 0.05±0.0008, 2 hrs: 0.21±0.001 a.u, 8 hrs: 0.4±0.001 a.u, 24 hrs: 0.47±0.008 a.u, 8 hrs+antalarmin: 0.04±0.0006, 24 hrs+antalarmin: 0.01±0.001). After showing that CRH had an upregulative effect on galectin-1 expression in macrophages, UCN peptide effect on galectin-1 expression had been tested at the same time points of stimulation (0, 2, 8, 24 hrs) in both cell types. UCN peptide caused an increase in galectin-1 expression mostly at 24 hrs of stimulation in macrophages (Figure 3b). (Galectin-1 expression in macrophages – UCN peptide stimulation for 0 hrs: 0.7±0.01, 2 hrs: 1.21±0.02 a.u, 8 hrs: 1.37±0.018 a.u, 24 hrs: 1.81±0.03 a.u).

## Discussion

In this study, we show for the first time that gal-1 is abundantly expressed in endometriotic tissue and that gal-1 is more highly expressed in eutopic endometrium of women with endometriosis compared to healthy women. Gal-1 was also found to be more highly expressed at late secretory phase of eutopic endometrium. Moreover, the neuropeptides CRH and UCN which are known to play a critical role in implantation and inflammation, were found to upregulate gal-1 expression by acting through the CRHR1 in Ishikawa cell line and macrophages.

### Galectin-1 is expressed in endometriotic sites and is overexpressed in ectopic sites compared to eutopic endometrium of endometriotic and healthy women. Galectin-1 is more abundantly expressed in eutopic endometrium of disease women compared to healthy ones

Galectin-1 has been reported to interfere with inflammation acting both as a pro- and an anti- inflammatory cytokine. Gal-1 has been strongly linked to cell growth and apoptosis of monocytes/macrophage [44]–[46] and was shown to induce apoptosis of activated immune cells. To this direction, galectin-1 has also been found to be involved in cell functions that characterize inflammatory responses such as in cell adhesion, chemotaxis and antigen presentation and apoptosis [47], [48] regulating the immune response. Of note is the fact that endometriosis is widely accepted as an inflammatory process: several proteins including growth factors, integrins, cadherins and lectins have been accounted for, being related to cell migration, invasion, angiogenesis, immune functions and apoptosis [49]. The anti-inflammatory properties of galectin-1 have been examined in several states of chronic inflammation and autoimmunity including autoimmune encephalomyelitis, arthritis, colitis, hepatitis, diabetes and chronic pancreatitis [50]. The finding of this current study, that galectin-1 is expressed in endometriotic sites, it is of high importance as it may regulate several immune functions concerning the pathogenesis of the disease and by being highly expressed in endometriotic sites, promoting T cell apoptosis, it could be hypothesized that gal-1 favors the persistence, establishment and immune escape of endometriotic tissue in ectopic sites. In addition, galectin-1 stimulates proliferation of vascular endothelial cells [51], so by being expressed in ectopic sites, it could hypothesized that it could facilitate the endometriotic tissue vascularogenesis, favoring the persistence of the disease. Moreover, in this study, it is found that galectin-1 is much more highly expressed in ectopic rather than eutopic endometrium of both endometriotic and healthy women and it could be hypothesized that galectin-1 decreased expression in eutopic endometrium compared to ectopic one of disease women indicates that its function outside the uterus, referring to adhesion formation and pain, might be strengthened. More experiments will be needed to clarify these hypotheses.

To further investigate the implication of gal-1 in reproduction defects of endometriotic women, we investigated galectin-1 expression in eutopic endometrium of endometriotic women compared to that of healthy women. We found that gal-1 expression is higher in eutopic endometrium of women with endometriosis compared to healthy ones. Taking into account the fact that gal-1 has been considered an important regulator of implantation our finding could indicate this increase of galectin-1 as a possible explanation to the endometriosis-related infertility. To this direction, the herein reported gal-1 upregulation of eutopic endometrium in case of endometriosis is in accordance with the established theory suggesting that several differences between eutopic endometrium of healthy women and endometriotic women have been correlated to infertility. These differences, apart from affecting fertility, are at the same time considered favourable for the endometrium to adhere, grow, and spread outside the uterine cavity [4], [52]. In addition, our finding expands the current perception upon galectins’ involvement in the pathophysiology of endometriosis, since until recently only galectin-3 had been shown to be significantly overexpressed in the eutopic endometrium of endometriotic women compared to the endometrium of healthy women [17].

We show also here that galectin-1 is expressed in endometrium at the late secretory phase and by decidual stromal cells. Decidualization and embryo implantation are affected by several endocrine, paracrine and autocrine factors such as hormones, cytokines and growth factors. As mentioned above, galectin-1 can act as an inflammatory cytokine involved in cell growth and macrophage function [47] [51] [44], [45]. However, the increased expression of apoptotic gal-1 in brain, placenta and other organs of the reproductive tract indicates that gal-1 might lead to T cell death, protecting those tissues from damage due to proinflammatory cytokines [51]. Galectin-1 has a similar expression pattern in placentas and extraembryonic membranes and it has also been linked to human trophoblast cell invasion mechanisms [16]. Additionally, galectin-1 being under the control of ovarian steroids [15], influences blastocyst implantation [53] and trophoblast invasion [54] mediating the maternal-embryonic immune/endocrine cross-talk, as well as placentation [16], [55]. The deranged gal-1 expression seems to negatively affect pregnancy outcome. Being highly expressed in human third trimester placentas and extraembryonic membranes, it is further upregulated in placentas of women with preeclampsia and in extraembryonic membranes in cases of chorioamnionitis. Interestingly, in abortions galectin-1 expression was reported to be decreased [56], with circulating gal-1 being marginally detected [54]. To the same direction, knock out mice of galectin-1 showed a higher rate of fetal loss in allogenic mating. All above published data together with our findings showing that gal-1 is differentially expressed in eutopic endometrium of endometriotic women and in eutopic endometrium of healthy women, suggest that gal-1 might be involved in implantation failure and thus infertility in case of endometriosis.

### CRH and UCN upregulate galectin-1 expression in Ishikawa cell line and macrophages. The CRH upregulative effect is mediated through CRHR1

We then examined how CRH and UCN regulate galectin-1 expression in an endometrial adenocarcinoma cell line, used as a eutopic epithelial endometrium model, aiming to further elucidate the role of these molecules in eutopic endometrium and infertility problems of women with endometriosis. We also examined these factors in macrophages which represent an important cell type in inflammation since endometriosis is a benign inflammatory disease. For the first time we show that CRH and UCN upregulate galectin-1 expression in Ishikawa cell line and macrophages and this effect is mediated through CRHR1.

The finding that CRH and UCN upregulate galectin-1 expression in Ishikawa cell line is of high importance since Ishikawa cell line is an *in* *vitro* model for endometrial endocrinology. That galectin-1 was found to be upregulated by neuropeptides that are expressed in eutopic and ectopic endometrium is also correlated with the finding showing that galectin-1 is overexpressed in endometriotic tissue. CRH, UCN have been found to be less expressed in eutopic endometrium of endometriotic women compared to ectopic endometrium [42]. In the same study, we also showed that CRH R1 and CRHR2 are significantly more expressed by the eutopic endometrium of women with endometriosis compared to the corresponding eutopic endometrium of healthy women [42], implying an increased CRH effect on the eutopic endometrium of women with endometriosis. To this direction, the significantly elevated gal-1 expression by the eutopic endometrium in case of endometriosis compared to healthy controls is thus in line with the proposed CRH-mediated gal-1 expression. The higher the effect CRH exerts on endometrial epithelial cells, the higher gal-1 expression is. Therefore it could be assumed that there is an autocrine or paracrine regulative network between CRH and gal-1 in the endometrium.

Galectin-1 is expressed in a variety of cells in central and peripheral immune sites including T cells, macrophages and activated B cells and modulates their functions and apoptosis [45], [51]. The fact that galectin-1 expression is upregulated in macrophages upon stimulation of CRH and UCN reveals new immunomodulatory roles of these cytokines in macrophages which are known to accumulate in endometriotic sites.

The upregulation of galectin-1 expression by CRH and UCN in macrophages indicates a new local gal-1-related immunomodulatory effect promoting a macrophage-mediated facilitation of endometriotic implants’ establishment by stimulation of endometrial cell proliferation, tissue remodeling and increased angiogenesis on the ectopic endometrial sites [57], [58]. Apart from that, gal-1 upregulation per se could lead to a locally increased apoptosis of activated T cells, contributing to local immune-escape and thus persistence of the endometriotic tissue.

In conclusion, it is the first time that galectin-1 has been shown to be overexpressed in endometriotic tissue. Galectin-1 showed an abundant expression in ectopic endometrium rather than in eutopic endometrium of endometriotic women suggesting that this protein could play a vital role in the pathogenesis of the disease. Moreover, gal-1 increased expression in eutopic endometrium of endometriotic women compared to healthy ones, could possibly indicate its implication in endometriotic women’s infertility profile, as gal-1 is a crucial immune factor involved in implantation and decidualization which expression is altered in a pathological condition such as endometriosis characterised by altered endometrium. CRH and UCN have been found to upregulate galectin-1 expression in Ishikawa cell line and macrophages. As these neuropeptides causing these upregulative effects and galectin-1 which is regulated by them are implicated in inflammatory procedures such as endometriosis and in reproductive functions, these results could possibly set new light to the immune disequilibrium of endometriosis and the infertility profile of endometriotic women. Finally, based on these results, showing that the CRH upregulative effect on galectin-1 is mediated by CRHR1, the potential use of Antalarmin could be reinforced in accessing the immune disequilibrium noticed in eutopic and ectopic endometrium of women with endometriosis.
